# Biomarkers in retinopathy of prematurity: a systematic review and meta-analysis

**DOI:** 10.3389/fped.2024.1371776

**Published:** 2024-03-20

**Authors:** Mariam Almutairi, Katherine Chechalk, Emelia Deane, Rebecca Fox, Ava Janes, Tidgh Maguire-Henry, Devin McCabe, Cole O'Connor, Joseph Quirk, Evan Swan, Katherine White, Kathryn McCreery, Eman Isweisi, Philip Stewart, Aoife Branagan, Edna F. Roche, Judith Meehan, Eleanor J. Molloy

**Affiliations:** ^1^ Discipline of Paediatrics, Trinity College Dublin, The University of Dublin, Dublin, Ireland; ^2^ Paediatric Ophthalmology, Children's Health Ireland (CHI) at Crumlin, Dublin, Ireland; ^3^ Paediatrics, Coombe Hospital, Dublin, Ireland; ^4^ Trinity Research in Childhood Centre (TRiCC), Trinity College Dublin, Dublin, Ireland; ^5^ Endocrinology, Children's Health Ireland (CHI) at Tallaght, Dublin, Ireland; ^6^ Trinity College Dublin, Trinity Translational Medicine Institute (TTMI), Trinity Centre for Health Sciences, St James Hospital, The University of Dublin, Dublin, Ireland; ^7^ Neurodisability, Children’s Health Ireland (CHI) at Tallaght, Dublin, Ireland; ^8^ Neonatology, Children's Health Ireland (CHI) at Crumlin, Dublin, Ireland

**Keywords:** retinopathy of prematurity, biomarker, metabolite, cytokine, growth factors, noncoding RNA

## Abstract

**Aim:**

Retinopathy of prematurity is a significant global cause of childhood blindness. This study aims to identify serum biomarkers that are associated with the development of ROP.

**Methods:**

A systematic review and meta-analysis was conducted using PRISMA guidelines. Three databases were searched (Pubmed, Scopus and Web of Science) from 2003 to March 2023. Only studies investigating serum biomarker levels in preterm infants (<37 weeks gestation) were included.

**Results:**

Meta-analysis suggests that low serum IGF-1 levels have a strong association with the development of ROP [SMD (95% CI) of −.46 [−.63, −.30], *p* < .001]. Meta-analysis suggests that higher serum glucose levels were associated with the development of ROP [SMD (95% CI) of 1.25 [.94, 1.55], *p* < .001]. Meta-analysis suggests that thrombocytopenia is associated with the development of ROP [SMD (95% CI) of −.62 [−.86, −.37], *p* < .001].

**Conclusion:**

Low levels of serum IGF-1, high levels of serum glucose and thrombocytopenia all appear to have the strongest association with the development of ROP out of the 63 biomarkers investigated in this review. These associations highlight their potential use as diagnostic biomarkers in ROP, though further research is needed to establish the exact relationship between these biomarkers and disease pathogenesis.

## Highlights

• This systematic review and meta-analysis evaluated the relationship between the levels of different serum biomarkers in preterm infants and the development of retinopathy of prematurity.
• In total, 66 studies investigating 63 biomarkers were included in the final review. These studies were generally of high quality, with a median score of 8 on the Newcastle Ottawa Scale.
• Low levels of IGF-1, high glucose levels and thrombocytopenia all appear to have the strongest relationship with development of retinopathy of prematurity.

## Introduction

Retinopathy of prematurity (ROP) is a proliferative retinal vascular disease that can occur in preterm infants (1). An estimated 50,000 children worldwide are blind due to ROP (2, 3), making it one of the leading causes of severe childhood blindness (4). ROP is a disease of prematurity and its incidence has risen as preterm survival has increased. Laser photocoagulation and anti-VEGF therapy are two of the main treatment options (5, 6).

Multiple risk factors contribute to the development of ROP including, longer duration of oxygen supplementation, pulmonary disease and low birth weight (1, 7). The pathophysiology of ROP occurs in two stages (8, 9). Retinal vasculature normally undergoes initial development at 4 months gestation *in utero (*9). When an infant is born prematurely, there is an abrupt cessation of retinal vessel maturation. This leads to retinal hypoxia as the limited vasculature does not meet the increasing metabolic demands of the proliferating retina. Disorganised neovascularization is then seen in response to this hypoxic insult. This revascularisation, combined with the ongoing hypoxia, damages the retina and can potentially lead to blindness (10). Understanding the complex relationship between inflammation and angiogenesis in the development of ROP provides a basis for investigating diagnostic biomarkers.

Various ROP screening programs exist worldwide involving early ophthalmology review identifying extent of retinal vasculature growth and degree of organisation denoted by the International Classification of Retinopathy of Prematurity (ICROP) staging (11), followed by continued monitoring. However, this requires significant time, energy, and skilled personnel, which may not be feasible, particularly in low and middle income countries (12). Screening criteria differs between nations. The United Kingdom guidelines, for example, recommend that all infants born less than 32 weeks' gestational age or under 1,501 g birth weight should be screened for ROP (13). Conversely, the US guidelines recommend screening for infants below 30 weeks' gestation or 1,500 g birth weight, or infants with a complicated postnatal course (14). While ROP is treatable through both surgical and medical approaches, the prognosis depends on disease staging (15).

The challenge remains in identifying infants at high risk of developing ROP and intervening early. An easily obtained, measurable and reliable marker of the disease that allows clinicians to achieve this isn't available to the authors knowledge. Serum is an easily accessible source of measurable compounds and can be obtained serially from the infant, causing minimal harm, by clinicians as needed. Staged progression of ROP as a disease suggests a temporally sensitive marker and identification of trends in measurements may be of value. This systematic review and meta-analysis aims to identify potential serum biomarkers in ROP to aid in early detection and therefore improved care for infants with ROP.

## Methods

### Search strategy

A systematic review was performed according to the Preferred Reporting Items for Systematic Review and Meta-analyses (PRISMA) guidelines (16). PubMed, Web of Science Core Collection and SCOPUS, were searched using the following term: “Retinopathy of Prematurity AND (biomarker OR metabolite OR cytokine OR growth factors OR noncoding RNA)”, identifying relevant literature from January 2003 until 22nd March 2023.

#### Inclusion and exclusion criteria

Inclusion criteria were generated according to PICO (Population, Intervention, Comparison, and Outcome).

The population was defined as preterm infants (neonates born before 37 weeks gestation) with and without a diagnosis of ROP. The intervention/exposure that was analysed was the presence of serum biomarkers in neonates with ROP. Serum biomarkers were defined as any naturally occurring amino acid, protein, sugar, or more complex biological structure that is suspended in either a neonate's blood plasma or present in cord blood at birth. The comparison was the difference between neonates with ROP and healthy neonates. Non-comparative studies which only investigated serum biomarkers in infants with ROP were also included. The outcome that was investigated was the potential link between these serum biomarkers and development or increased severity of ROP in preterm infants.

**Figure 1:**
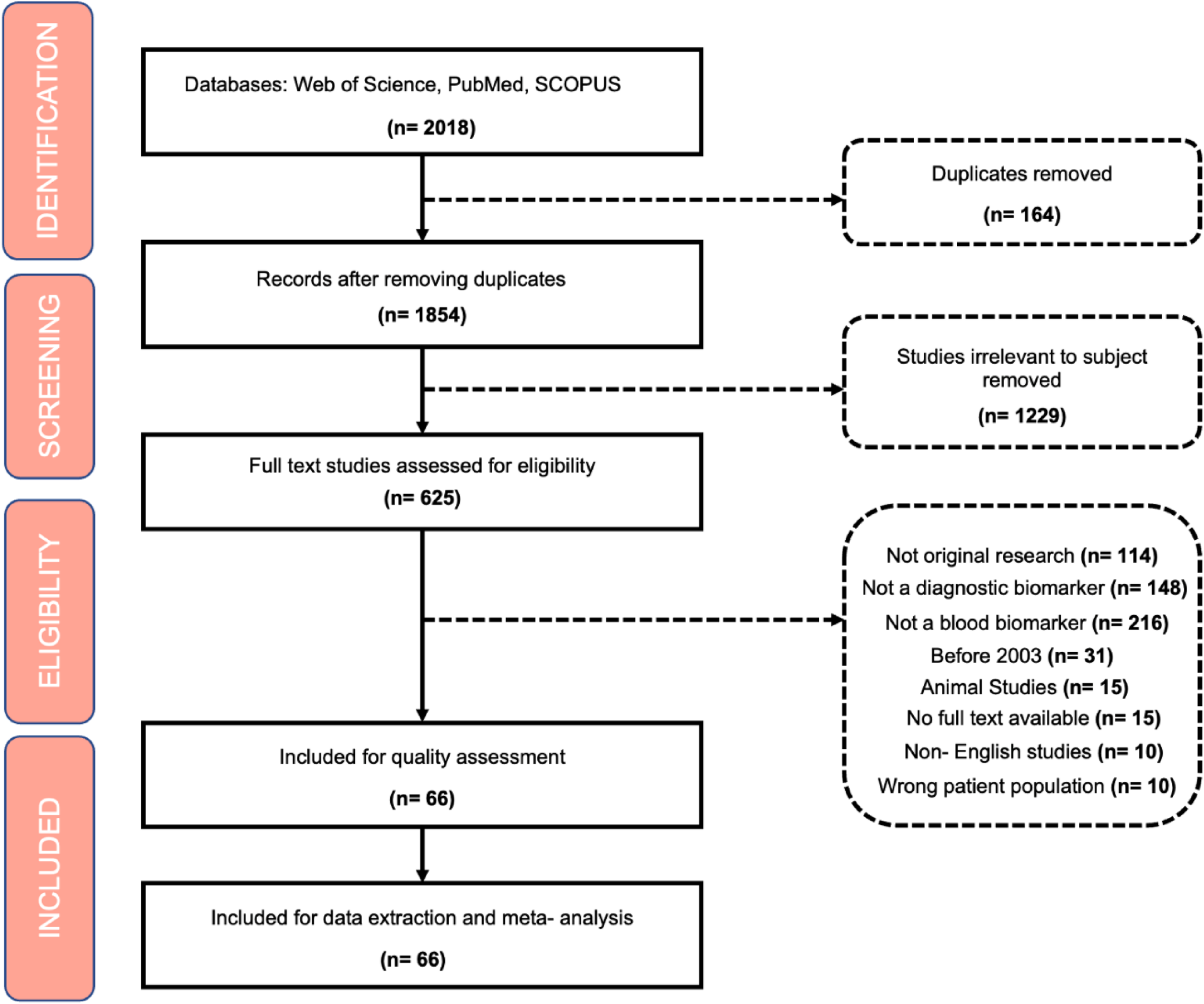
Resulting PRISMA flow of the systematic literature search strategy.

Exclusion criteria included: (1) Non-primary research, literature reviews, letters to the editor; (2) animal studies; (3) Non-diagnostic biomarkers; (4) studies published prior to 2003; (5) non-blood biomarkers; (6); (7) duplicate articles, 8) non-English studies (Figure 1).

### Screening

All studies identified were exported to Covidence (16) where duplicates were automatically removed. Articles were initially screened for title, abstract and keywords related to the subject of biomarkers in ROP. All papers were screened individually by two independent reviewers and disagreements were resolved by a third reviewer.

In total 2018 papers were extracted from the search strategy. Then, 1,352 articles were removed if they did not meet the inclusion criteria or if they were duplicates. In total, 666 articles met the inclusion criteria and were ultimately included for review and meta-analysis.

#### Quality assessment

Reporting quality of all included studies was assessed using the Newcastle Ottawa Scale. (NOS) checklist for case-control and cohort studies. Each study was appraised following 3 parameters: selection, comparability and outcome; worth four, two and three points, respectively. Good quality studies were defined as having 3 or 4 stars in the selection domain and 1 or 2 stars in the comparability domain and 2 or 3 stars in the outcome/exposure domain (17).

#### Statistical analysis

Only biomarkers investigated in 2 or more studies and comparative quantitative studies that reported mean and standard deviation were included for pooling in meta-analysis. These values were used to estimate standard mean difference (SMD) with a 95% confidence interval, and heterogeneity across studies on individual biomarkers using RevMan version 5.4 statistical software (18). For certain studies that reported a median and range, data were transformed to mean and standard deviation according to formulae reported by Hozo et al. (19) *P* < 0.05 was considered statistical significant.

The combination of results was done using the random- effects model in heterogeneous studies and fixed-effects model in homogeneous studies. SMDs were used as common measures for biomarker measurements in ROP vs. non-ROP neonates. Publication bias was assessed by visual inspection of Begg's funnel plots.

## Results

The selected papers included 35 cohort studies, 26 case-control studies, 4 metabolomic studies and 1 chart review.

### Quality assessment

The median quality score was 8 and the range was 6–9 according to the Newcastle-Ottawa scale for reporting.

#### Biomarkers investigated

In total, 63 possible biomarkers for predicting ROP were identified (Table 1).

**Table 1:** Identified biomarkers following systematic review.

Biomarker investigated	Number of studies
IGF-1, VEGF	17
Glucose	10
Platelets	7
IL-6	5
TNF-alpha, VEGFR-1	4
IL-8, VEGF-A, apelin, EPO	3
Creatinine, citrulline, IGFBP-3, VEGFR-2, sE-selectin	2
Glycated albumin, ADMA, HIF-alpha, Ang 1, Ang 2, BUN, BDNF, PDGF-BB, PEDF MPO, ICAM 1, MMP-9, bFGF, CD133, CD34, CD144, Foetal haemoglobin, MnSOD, CRDL1, PCSK9, Glycine C3DC, Arginine, Aminoadipic acid, IL-7, MCP-1, MIP-1a, MIL-1b, Haemoglobin A1c, Prolactin, Vasoinhibins, IL-5, RANTES, C5A, HSA circle RNAs, HTRA-1, TGFB-1, TGFB-2, sTie2, Nfl, GFAP, Endostatin, Peptides, Proteins, Proline, Ribitol, Glutamic acid, Gamma methyl ester	1

### IGF-1

IGF-1 was investigated in 17 studies. Lower IGF-1 levels are associated with the development of retinopathy of prematurity according to 9 studies. No statistically significant association with serum IGF-1 levels and ROP was noted in 7 studies. A single study found that fluctuations in IGF-1 levels were associated with the development of retinopathy of prematurity.

**Figure 2:**
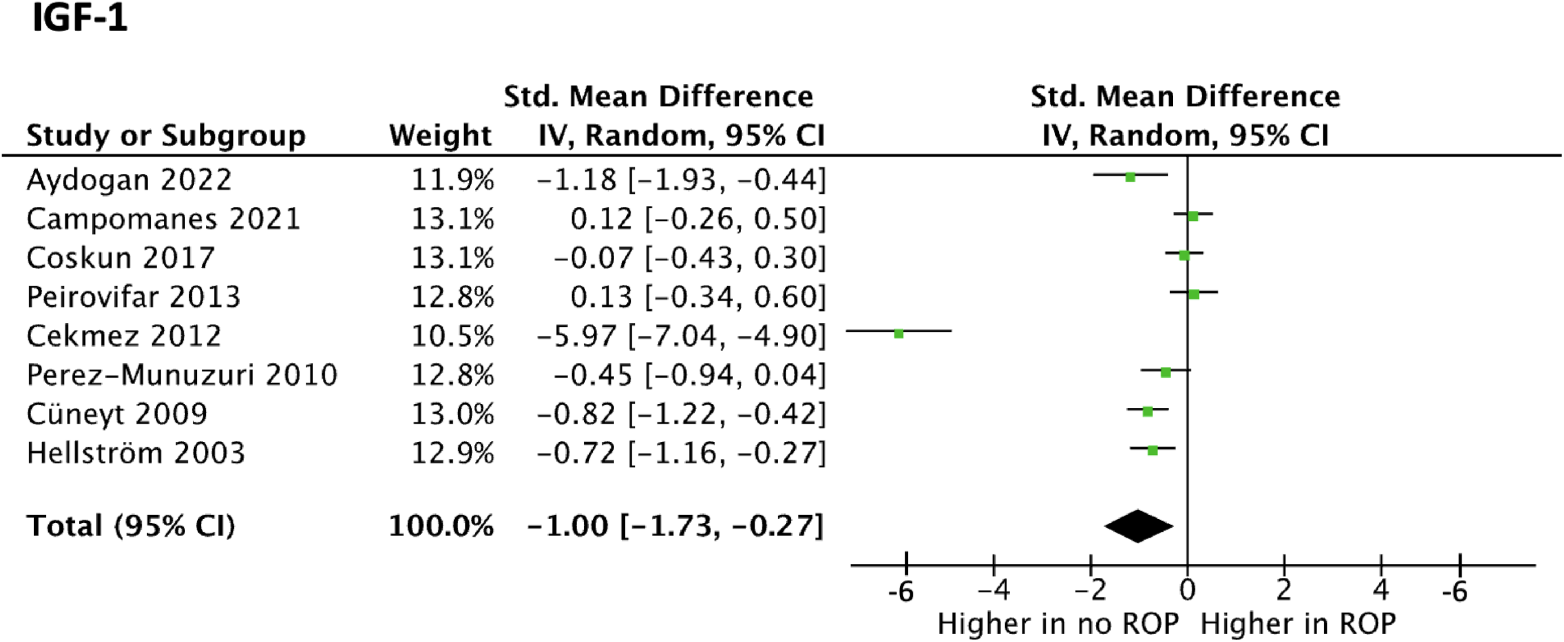
Forest plot of the standard mean difference (SMD) of IGF blood levels in neonates with and without ROP; and 95% confidence interval based on random effect model in meta-analysis.

A total of 8 studies investigating IGF-1 were suitable for meta-analysis. Meta-analysis revealed statistically significant data (*p* value < 0.01) with a SMD [95% confidence interval] of −0.46 [−0.63, −0.30] as seen in Figure 2. This suggests IGF-1 levels may be greater in preterm infants without ROP compared to controls.

### VEGF

**Figure 3:**
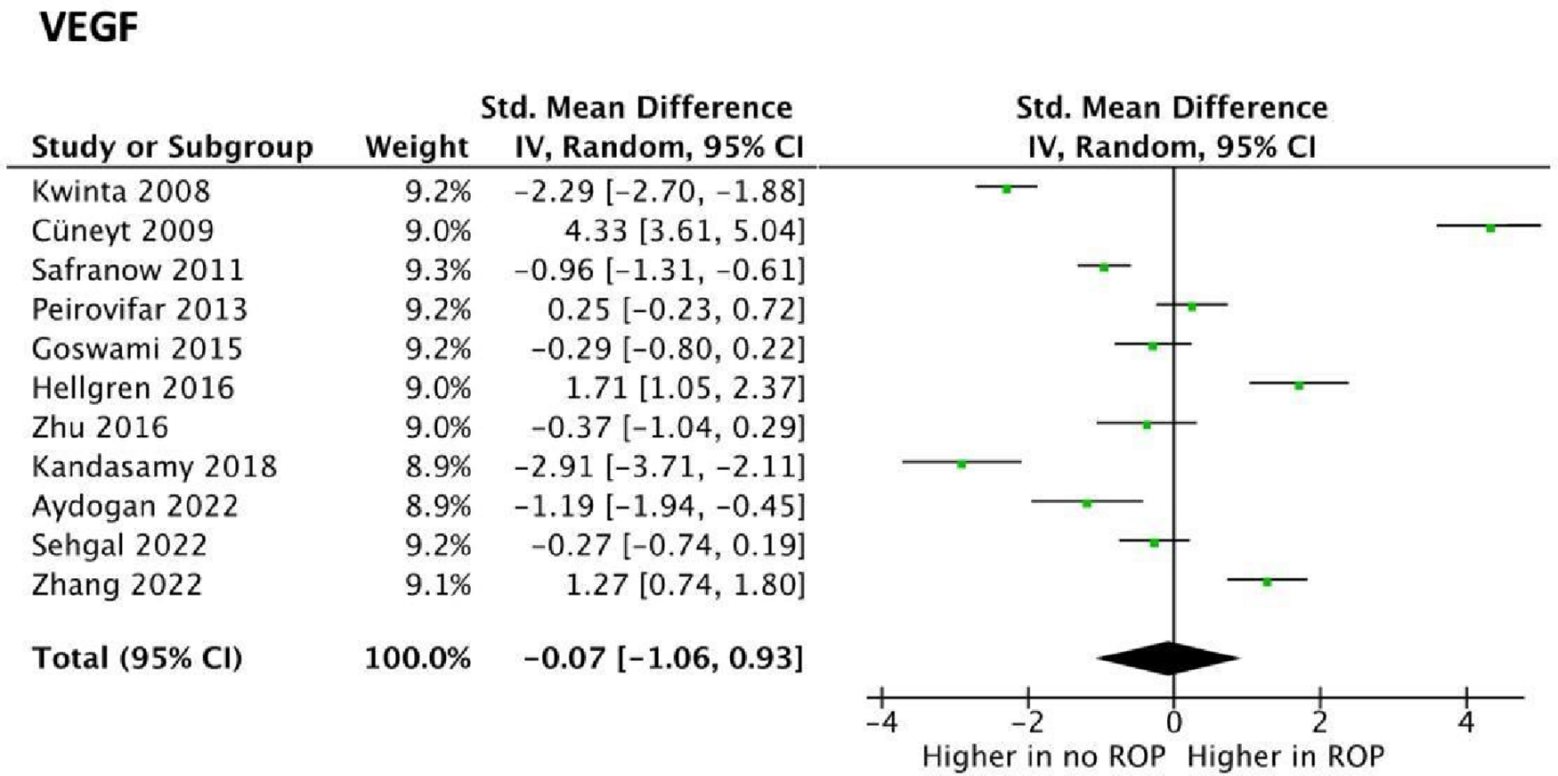
Forest plots of the standard mean difference (SMD) of VEGF blood levels in neonates with and without ROP; and 95% confidence interval based on random effect model in meta-analysis. The midpoint of each segment, the segment estimating the SMD, and 95% confidence interval in each study are shown Diamond mark overall SMD based on results of the meta-analysis.

VEGF was explored as a potential biomarker in 17 studies, all of which utilised ELISA. Of these, 8 studies reported higher serum VEGF levels associated with the development of ROP. No statistically significant difference in serum VEGF concentrations in infants who did not have ROP and those who did was found in 9 studies. VEGF levels in ROP were suitable for meta-analysis in 11 studies. Meta-analysis revealed statistically significant data (*p* value <001) with a SMD [95% confidence interval] of −0.07 [−1.06, 0.93] as seen in Figure 3. This suggests VEGF may be lower in preterm infants with ROP. However, studies had highly heterogeneous results (*I*^2^ = 97%) and, more importantly, the confidence interval crossed the neutral line in Figure 3. These findings both suggest that no conclusions can be drawn on whether VEGF levels in fact differ in premature infants with or without ROP.

VEGFR-1 and VEGFR-2 were discussed in 4 and 2 studies, respectively. All 6 studies evaluated using ELISA. No statistically significant difference between VEGFR-1 and 2 levels were identified in the development of ROP.

### Glucose

Serum glucose was discussed in 10 studies, of which 8 studies measured glucose using a glucometer, 1 using the glucose oxidation technique, 1 study using full blood count. All studies found a statistically significant correlation between raised serum glucose in mothers and infants for the development of ROP.

Three studies reported on serum glucose levels in ROP were suitable for meta-analysis. Meta-analysis revealed statistically significant data (*p* value <0.01) with a SMD [95% confidence interval] of 1.25 [0.94, 1.55] Table 2. This suggests glucose levels are greater in preterm infants with ROP compared to those without. Results across the studies were relatively homogenous (*I*^2^ = 56%).

**Table 2:** Standard mean difference (SMD) of biomarker blood levels in neonates with and without ROP and 95% confidence interval (CI) from meta-analysis. The number of studies that reported on each biomarker, the sample size, overall effect, and heterogeneity from meta-analysis.

					Heterogeneity	
Biomarker	Number of studies	Sample size	SDM (95% confidence interval)	Overall effect (*p* value)	*I* ^2^	*P* value
VEGF	11	873	−0.33 [−0.49, −0.18]	<0.0001	97%	<0.00001
IGF-1	8	696	−0.46 [−0.63, −0.30]	<0.00001	95%	<0.00001
Glucose	3	587	1.25 [0.94, 1.55]	<0.00001	56%	0.11
IL-6	3	255	0.5 [0.22, 0.77]	<0.0004	94%	<0.00001
IL-8	2	145	0.98 [0.61, 1.35]	<0.00001	95%	<0.00001
Platelets	2	277	−0.62 [−0.86, −0.37]	<0.00001	95%	<0.00001
Endostatin	2	120	0.05 [−0.35, 0.46]	0.79	66%	0.09
Apelin	2	154	−2.13 [−2.59, −1.67]	<0.00001	98%	<0.00001
sE-selectin	2	88	1.64 [1.13, 2.15]	<0.00001	0%	0.07

### Platelets

Platelets were investigated in 7 studies, 6 of which used full blood count, while 1 study evaluated using immunoassay. All studies revealed that thrombocytopenia in premature infants has a considerable relationship with the development of ROP. Thrombocytopenia was defined as a platelet count of less than 150 × 10^(9)^/L. Two studies reporting on platelet levels in ROP were suitable for meta-analysis. Meta-analysis revealed statistically significant data (*p* value <0.01) with a standard mean difference [95% confidence interval] of −0.62 [−0.86, −0.37] Table 2. This suggests platelet levels are decreased in preterm infants with ROP compared to those without ROP. While results suggest decreased platelet levels are found in ROP, many studies similarly reported on this as thrombocytopenia as a risk factor for ROP. Therefore, a meta-analysis that pools this dichotomous data and reports on outcome ratios may be suitable.

### Metabolomics

Metabolomic analysis was used to identify potential metabolic biomarkers in 4 studies (20–23), which measured individual concentrations of metabolites.

Metabolomic studies involving liquid chromatography and mass spectrometry demonstrated an increase in the following metabolites: Citrulline, Arginine, Aminoadipic acid, Proline, and Creatinine. Elevated Citrulline, Arginine, Aminoadipic acid as well as decreased serum plasma creatine were associated with ROP.

Two studies identified additional potential biomarkers. Glycine, Glutamate, Leucine, Valine and Homocysteine appear to be promising biomarkers for the prediction of occurrence rather than severity of ROP (22, 23).

IL-6, IL-8, apelin and sE-selectin were found to be associated with ROP in preterm infants. Meta-analysis revealed statistically significant data (*p* < 0.01) with a SMD [95% confidence interval] of 0.50 [0.22, 0.77] and 0.98 [0.61, 1.35] for IL-6 and Il-8 levels, respectively (Table 2). This suggests that levels of these interleukins are increased in those with ROP. The same can be said for sE-selectin which had a SMD [95% confidence interval] of 1.64 [1.13, 2.15] across studies. Conversely, meta-analysis revealed statistically significant data (*p* value <0.01) finding decreased levels of apelin in the blood is associated with ROP; SMD [95% confidence interval] of −2.13 [−2.59, −1.67].

#### Publication bias

**Figure 4:**
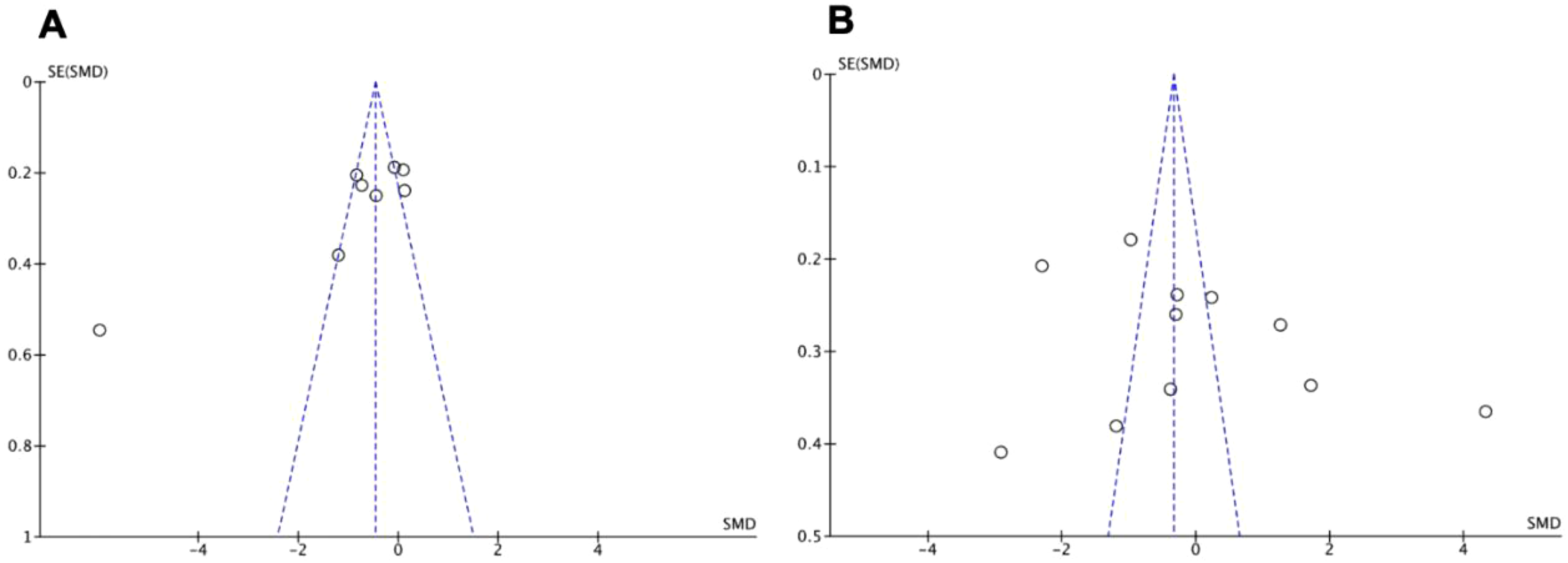
Funnel charts for studies included in meta-analysis for (**A**) IGF-1 and (**B**) VEGF are shown.

Visual inspection of funnel plots was performed to assess for publication bias. The funnel plots were symmetrical, as seen in Figure 4 for the two most studied biomarkers, indicating absence of publication bias.

## Discussion

ROP remains a major cause of acquired blindness in children despite being a treatable disease. Serum biomarkers offer a less invasive and more widely applicable approach to diagnosing ROP, with the potential to detect disease processes before symptoms have manifested. In this study, proposed infant and cord blood serum biomarkers from the past 20 years were reviewed, which resulted in the identification of 63 potential biomarkers. Statistically significant differences were found in 9 biomarkers suitable for meta-analysis, however issues with heterogeneity between studies interfered with the formation of a conclusive result for some. In general, it was determined that several biomarkers are likely involved in ROP with IGF-1, VEGF, and glucose being studied in the greatest depth. All three of these biomarkers share particularly promising diagnostic and prognostic significance. In addition, other haematological and biochemical markers were identified which may provide a useful adjunct. The specific results for individual biomarkers will now be discussed in further detail.

### Cytokines/growth factors

#### IGF-1

IGF-1 was proposed as a possible biomarker due to its role in the development of the foetal retina and therefore is likely implicated in the pathogenesis of ROP. A successful biomarker for ROP must be practically useful and sensitive in diagnosing to replace non-invasive measurements such as gestational age, birth weight and head circumference.

Notable findings included 7 studies with no significant differences between the ROP and no-ROP cohort, while 9 studies found the ROP cohort to have a lower IGF-1 level. With no timing standardisation, discrepancies arose, possibly explaining the range of serum concentrations between the two cohorts. Aydoğan et al. report no significant difference in serum IGF-1 levels between preterm infants with and without ROP (24). However, Perez-Munuzuri et al. found that IGF-1 levels were significantly lower at the 3rd week post-partum in the group that developed ROP, independent of gestational age at birth (25). The authors propose a threshold value of 30 ng/ml IGF-1 at the 3rd week postpartum that has 90% sensitivity for the diagnosis of ROP. Reddy et al. demonstrated using logistic regression that the chance of developing ROP reduced for every unit increase in serum IGF-1 between 31 and 33 weeks. Hellström et al. report that persistent low IGF-1 levels in preterm infants from birth were correlated with both the development and severity of retinopathy of prematurity (26). The concentration of IGF-1 in the neonate seems to fluctuate rapidly over the first few weeks of life, and so going forward, there must be standardised timing of sampling, relative to the neonates' postpartum age (24, 25). Further studies with increased sample size will be needed to confirm whether fluctuations and trends in a neonate's IGF-1 concentrations will be a useful prognostic or diagnostic tool.

#### VEGF

Vascular Endothelial Growth Factor (VEGF) is an important signalling protein involved in both angiogenesis and vasculogenesis (27). VEGF concentrations showed mixed results regarding its predictive value as a biomarker in ROP (Figure 3).

Hellgren et al. (28) described varying VEGF concentrations depending on the timepoint VEGF was obtained from the infant (29). This represents an important distinguishing feature as relevant physiological variation that may contribute to the pathogenesis of ROP, with no significant difference in VEGF at the time of birth, though differences were noted over time (27). Thus, as no standardised timing for sample collection exists between compared studies, the robustness of the meta-analysis is inherently limited in the present article. Similar to IGF-1, discrepancies were noted between the source of each sample analysed, with a fraction of studies evaluating umbilical cord-derived blood (30) while others analysed serum samples (31). This variation may further limit the predictive value of VEGF when comparing studies as a group. For example, lower VEGF concentrations in infants with ROP were found in umbilical cord-blood compared to serum derived VEGF samples (31). Similarly, umbilical cord-blood VEGF levels were found to be independently associated with subsequent development of retinopathy of prematurity (31). Therefore, both timing of VEGF measurement as well as sample source represent two areas of further refinement, suggesting a need for standardisation in future studies to elucidate the distinct role of VEGF.

Similarly, vascular endothelial growth factor receptors 1 and 2 (VEGFR-1, VEGFR-2) were analysed in 6 studies using Enzyme-linked immunosorbent Assay (ELISA). ELISA uses the principle of tagging antigen-antibody reactions to select for specific cytokines, proteins, peptides, and other biomarkers of interest. ELISA is a relatively cost effective, safe, and simple procedure to perform but requires skilled lab technicians and has a high possibility for false results with the added effort to keep the antibody in ideal conditions (32). VEGFR-1 demonstrated no statistically significant predictive value in ROP (33). While VEGFR-2 s demonstrated opposing results (34) with otherwise similar methods. Thus, as ELISA may be subject to some inherent variability by nature of the assay, further analysis using standardised methods may be of value in determining the role of VEGFR-2 in the pathogenesis of ROP.

#### IL-6, Il-8—TNF-aIpha and GFPB-3

Elevated levels of proinflammatory cytokines; IL-6, 8, Tumour Necrosis Factor-Alpha (TNF-a) and significantly lower levels of IGFBP-3 have been associated with the development and progression of ROP (27). It was found that umbilical cord concentrations of TNF-a and IL-6 within the first few days of life were significantly elevated in those with ROP stage(s) 2–4 (15) compared to those with stage 1 ROP. Sileria et al., supported these findings as increased pro-inflammatory cytokines 72 h post birth were associated with a higher incidence of developing ROP. Similarly, Stood et al., reporting that infants who had been treated for ROP had an elevated level of IL6 at birth and up to 24 h after. These individual studies of collected data, concluding that, there may be a relationship between elevated circulating inflammatory biomarkers within the early hours of birth (<72 h) that have an association with the later development of ROP.

### Haematology/biochemistry

#### Platelets

The potential relationship between platelet counts and the development of ROP was analysed in 7 studies. While not fully understood, thrombocytopenia during the important period of retinal development after preterm birth is associated with ROP (27, 29, 35–39). Both Hellgren et al. and Cakir et al. showed that platelet-released factors and thrombocytopenia affect the regulation of retinal and systemic angiogenesis in premature infants (27, 29). Thrombocytopenia during the neovascularization stage is consequently associated with the development of severe ROP in premature infants.

The link between thrombocytopenia with ROP indicates that platelets possess proangiogenic effects in the normal retinal vasculature development (29). This is clinically significant as monitoring platelet count is minimally invasive but provides care teams with insight into potential complications (37).

#### Erythropoietin (EPO)

Erythropoietin (EPO) is a peptide hormone produced by the kidney which promotes erythrocyte differentiation and maturation (40). Three studies analysed EPO in the context of inflammation, often being measured in association with VEGF and IGF-1 (31, 40–42). While none of the studies could conclude if EPO was associated with ROP in isolation, it was linked to low levels of VEGF (12) which historically is a more likely biomarker for ROP. One anomaly that recurred was the lower levels of EPO in babies with ROP. This is remarkable as EPO is typically activated in hypoxic states (31), however despite hypoxia being a common problem for premature babies, the measurements of EPO reported lower (40). EPO demonstrates promise as a biomarker, however an isolated study would be required to make any definite conclusions.

#### Glucose

Hyperglycaemia in the premature infant is a common problem, with incidence estimated between 45% and 80% in the extreme low birth weight (ELBW) preterm infant (43). Neonates in the early postnatal period do not have well developed regulatory systems for glucose and thus are more susceptible to serum glucose concentration derangements. In prematurity, immature insulin production and physiological insulin resistance of prematurity can lead to dysregulated glucose homeostasis (44).

This review included 10 papers that investigated the relationship between serum glucose levels and the development of ROP. Studies measured glucose either by glucometer or serum glucose concentrations. All studies except one used dichotomous data, defining hyperglycaemia with a value of greater than 150 mg/dl. The other study used a cumulative, time-weighted glucose level (TWGL). Using 10 days of TWGL greater than 100 mg/dl, and 30 days of TWGL greater than 118 mg/dl. One study additionally measured the mean serum glucose level in neonates with and without ROP. All the papers conclude that the incidence of ROP is significantly higher in premature infants who develop hyperglycemia. However, the exact nature of this link remains somewhat elusive.

Chronic hyperglycemia in adults in the setting of diabetes mellitus is associated with a myriad of conditions, including diabetic retinopathy (45). Like ROP, diabetic retinopathy is underpinned by a proliferative vascular mechanism of disease, which can lead to retinal detachment (46). Hyperglycaemia and ROP have a clear association, but it remains unclear whether this relationship is causal or not. However, the similarities between diabetic retinopathy and ROP, and the importance of glucose in the pathogenesis of diabetic retinopathy, suggest that there may be a link between the two that warrants further investigation.

The data in the studies included in this review point to hyperglycaemia being both an independent risk factor for ROP (38, 47, 48) and a marker of severity of disease. It has been shown that it is overall average exposure to high glucose concentrations that is associated with ROP, rather than single episodes of hyperglycaemia. The number and severity of hyperglycaemic events also correlates with increased incidence of both mild and severe ROP (47, 49), suggesting that the association is dose dependent.

For its use as a biomarker, further research may be needed to elucidate the exact nature of the relationship between hyperglycaemia and ROP. It has been hypothesised that this association is because of glucose itself on vasculature, or that it is underpinned by decreased IGF1 expression occurring secondary to decreased insulin signalling. Additionally, thresholds of severity and duration of hyperglycemia were identified in one paper, above which the risk of ROP increased significantly (47), however there is little other research on this topic, and further investigation is needed to clarify above which glucose concentrations the risk of ROP increases.

Glucose could potentially be utilised as both a predictive biomarker and as a biomarker of severity in the setting of ROP. Additionally, as it appears to be an independent risk factor for ROP, blood glucose testing could also have a role in its prevention. However, further investigation into its relationship with ROP with the view of using it as a biomarker should be conducted to create a structured approach to its use.

### Metabolomics and other biomarkers

#### Metabolites

The retina is highly metabolically active given its large energy demands, and a link between metabolic changes and retinal vasculopathy has previously been established (50). For this reason, it was surprising to find few studies that explored the use of metabolomics to identify ROP biomarkers. Of the studies included in our review, all found a correlation between metabolic perturbation and the pathological mechanisms seen in ROP.

Abnormal retinal angiogenesis, neovascularization, and some altered metabolites serve as candidate biomarkers for the diagnosis of ROP. Of these, citrulline and creatinine demonstrated a statistically significant difference between ROP and non-ROP groups in more than one study (20, 41). The upregulation of citrulline in ROP groups is supported by prior research regarding ischaemic retinopathies (IRs) (20, 21) which note that through its involvement in the arginine-citrulline pathway, the metabolite has a role in nitric oxide mediated alteration of retinal pigment epithelium and angiogenesis (51). A promising finding in Yang et al.'s study (22) indicated that some biomarkers had the potential to be detected before symptoms emerged. Despite such results, longitudinal studies with larger sample sizes are recommended to further investigate the causal relationship between these altered metabolites and ROP, and therefore, their significance in the clinical setting (52).

### Apelin

This angiogenic factor forms a part of the apelin-APJ system, which is highly expressed in vascular endothelial cells and is known to play a role in the processes involved in various IRs, such as retinal angiogenesis. However, research surrounding the correlation between serum apelin levels and ROP has so far been inconclusive, with opposing results found between different studies. While Zhang et al. and Cekemez at al. (48, 52). both found plasma apelin levels to be significantly lower in infants with ROP as compared to those without ROP, the opposite was noted by Feng et al. (41) Furthermore, this latter study found plasma apelin levels to positively correlate with ROP severity, which has also been seen in diabetic retinopathy (reference), therefore suggesting that apelin might aggregate pathological processes and be a candidate for predicting disease severity, rather than occurrence.

Discrepancies in population characteristics, including gestational age and sample evaluation times, are suggested to play a role in the differing results between the studies (41). This is an important finding that requires further exploration as, similarly to VEGF, this indicates that the pathogenesis of ROP may be linked to a relevant physiological variation is required to further elucidate the correlation and causality between serum apelin levels and ROP, considering the above-mentioned discrepancies as well as ensuring multi-centre involvement and larger sample sizes.

### sE-selectin

Similarly to apelin, selectins have recently been implicated in angiogenesis, with elevated levels of E-selectin being present in both ocular and non-ocular vaso-proliferative disorders, such as diabetic retinopathy, rheumatoid arthritis, and tumour growth (34, 53). The soluble form of E-selectin (sE-selectin) found in plasma correlates with its expression on endothelial cells, and in both studies included in our review, serum sE-selectin levels were found to be significantly higher in infants with ROP as opposed to those without ROP (references). Indeed, a causative link between sE-selectin and the pathogenesis of ROP is supported by the longitudinal results of the study by Peih et al. (53), as these elevated sE-selectin levels seen in infants with ROP were constant at all time points, suggesting that sE-selectin may influence the development of ROP. Furthermore, results from both studies suggested that serum sE-selectin levels have the potential to identify preterm infants at risk of ROP within two days of life, a finding of significant clinical promise. To date, however, there have been few studies exploring the relationship between sE-selectin and ROP. Therefore, to confirm and enhance the value of the above findings, further prospective studies with larger population sizes are recommended.

## Strengths and limitations

### Strengths

The scope of the systematic review was clear, with the review being conducted across multiple databases and with a predefined inclusion/exclusion criteria for study design, population, exposure, comparators, and outcomes. Screening and full text review of studies were performed independently by two separate groups of reviewers, with any conflicts of opinion being resolved by a third-party reviewer. Data extraction was performed independently, and papers were excluded where the investigators of the primary studies were unable to provide required data following an attempt at contact. The reporting of the search strategy and subsequent screening and review of studies followed the requirements of the PRISMA statement. A quality assessment of the evidence for the main analyses was carried out using the Newcastle-Ottawa Scale (NOS). Studied biomarkers are easily obtained from minimally invasive blood sampling. Inflammatory cytokines and growth factors were identified to be strong possible biomarkers for ROP, findings revealed temporal sampling of these serum biomarkers by clinicians can identify those at risk. Recommendations for future studies on these biomarkers to allow for more clinically translatable findings are given. This novel study encompasses findings from available human studies on serum biomarkers in ROP, which has yet to be reported on systematically.

### Limitations

No assessment of the risk of study selection bias was performed. Heterogeneity involving population, ROP severity and use of a control existed between studies. Meta-analysis of data in some studies required an approximation of mean and standard deviation where only median and range were given (19). While results from meta-analysis suggest increased glucose and decreased platelet levels are found in those with ROP, many studies reported similar results but as hyperglycaemia and thrombocytopenia, respectively, as risk factors for ROP. Therefore, a meta-analysis that pools this dichotomous data from these studies and reports on outcome ratios may be suitable and yield more data.

## Conclusion

The lack of homogeneity between screening recommendations, combined with the labour-intensive process of current diagnosis reflects the complex nature of ROP and the necessity for diagnostic biomarkers. Several biomarkers have been identified, with IGF-1, VEGF, and glucose having the greatest number of associated studies, all of which share promising diagnostic and prognostic significance. Further study is still required to define each biomarker's mechanism of action in ROP as well as identify consistent extraction and isolation methods. Findings from this study can be used as a valuable resource for future clinical researchers to define their methodology and encourage universal practises when reporting.

## Data Availability

The original contributions presented in the study are included in the article/Supplementary Material, further inquiries can be directed to the corresponding author.
